# Adenylate cyclase toxin of *Bordetella parapertussis* disrupts the epithelial barrier granting the bacterial access to the intracellular space of epithelial cells

**DOI:** 10.1371/journal.pone.0291331

**Published:** 2023-11-27

**Authors:** Juan Pablo Gorgojo, Mariela del Carmen Carrica, Carlos Manuel Baroli, Hugo Alberto Valdez, Jimena Alvarez Hayes, Maria Eugenia Rodriguez

**Affiliations:** CINDEFI (UNLP-CONICET), Facultad de Ciencias Exactas, Universidad Nacional de La Plata, La Plata, Argentina; CRCL: Centre de Recherche en Cancerologie de Lyon, FRANCE

## Abstract

*B*. *parapertussis* is one of the etiological agents of whooping cough. Once inhaled, the bacteria bind to the respiratory epithelium and start the infection. Little is known about this first step of host colonization and the role of the human airway epithelial barrier on *B*. *parapertussis* infection. We here investigated the outcome of the interaction of *B*. *parapertussis* with a polarized monolayer of respiratory epithelial cells. Our results show that *B*. *parapertussis* preferentially attaches to the intercellular boundaries, and causes the disruption of the tight junction integrity through the action of adenylate cyclase toxin (CyaA). We further found evidence indicating that this disruption enables the bacterial access to components of the basolateral membrane of epithelial cells to which *B*. *parapertussis* efficiently attaches and gains access to the intracellular location, where it can survive and eventually spread back into the extracellular environment. Altogether, these results suggest that the adenylate cyclase toxin enables *B*. *parapertussis* to overcome the epithelial barrier and eventually establish a niche of persistence within the respiratory epithelial cells.

## Introduction

*B*. *parapertussis* and *B*. *pertussis* are the etiological agents of whooping cough, the worst controlled vaccine-preventable disease that has reemerged even in highly vaccinated populations [1]. Infections by *B*. *parapertussis* significantly contribute to the overall pertussis burden and reemergence [2–4]. Since the introduction of acellular vaccines, the incidence of this bacterium has been increasing globally [2, 5, 6]. The rise in *B*. *parapertussis* cases was mainly attributed to the failure of acellular vaccines to control this pathogen. Although acellular vaccines are formulated with *B*. *pertussis* antigens also expressed by *B*. *parapertussis*, they do not protect against this latter bacterium [7] likely because of the expression of the O antigen by *B*. *parapertussis*, which blocks antibody access to surface antigens [8], and the antigenic divergence between the species [9, 10]. The lack of cross-immunity enables *B*. *parapertussis* to avoid bactericidal mechanisms induced by vaccine antibodies [11–14]. We previously found evidence demonstrating that, in the absence of specific antibodies, *B*. *parapertussis* survives the innate interaction with neutrophils and macrophages. In these cells phagocytosed bacteria efficiently preclude phagolysosomal fusion and remain viable intracellularly [11, 12], suggesting that *B*. *parapertussis* might have an intracellular stage inside the host cells likely contributing to the establishment of persistent infections.

Airway epithelial cells are the main cell type that inhaled bacteria encounter once in the host. These cells play a crucial role in the host defense against invading bacteria. However, they eventually develop into an intracellular niche of persistence for respiratory pathogens [15]. The bacterial ability to invade this kind of cells, adapt to the intracellular environment, and survive within them, usually leads to the development of chronic infections [15]. Respiratory epithelial cells form a polarized layer that provides both signaling and physical barrier functions against inhaled pathogens. The preservation of the protective role of the epithelial layer depends on the integrity of the tight junctions. These structures keep the cells strongly attached to each other regulating the barrier function of the epithelium while contributing to cell polarity by blocking the free diffusion of molecules between two well-defined domains, the apical and basolateral membranes [16]. These membranes contain different sets of sorted molecules and proteins, resulting in membranes with different functions [17]. The differential expression of molecules in these membranes usually determines the affinity of the pathogen for one or the other [18]. While several pathogens directly attach to the exposed apical membrane, others preferentially bind to target sites in the basolateral membrane [19–22]. The bacterial access to these preferred binding sites eventually takes place by a gap in the tight junctions, which may be induced by bacterial virulence factors [23–25], during PMN transmigration to the site of infection [26], or during cell division and/or cell extrusion [27, 28].

In the present study we explored the interaction of *B*. *parapertussis* with a monolayer of polarized respiratory epithelial cells and the potential role of this type of cells in the bacterial persistence within the host

## Materials and methods

### Bacterial strains and growth

The *B*. *parapertussis* strain CN2591 [29] and an isogenic *B*. *parapertussis* mutant strain lacking adenylate cyclase toxin (BppΔcyaA) [30], were used in this study. Bacteria were stored at -70°C and recovered by growth on Bordet-Gengou agar (BGA) (BD Difco, Sparks, MD) plates supplemented with 15% defibrinated sheep blood (Laboratorio Argentino, Caseros, Argentina) for 24 h at 36°C. Animal handling and all procedures were in compliance with the Argentinean animal protection Law 14346. The study was approved by the Institutional Animal Care and Use Committee (CICUAL) of Faculty of Sciences, National University of La Plata. Bacteria were subsequently seeded on Stainer-Scholte liquid medium, cultured for 20 h at 36°C, and used in the experiments.

### Antibodies

The following antibodies were used in this study: mouse monoclonal antibodies against human lysosome-associated membrane protein 1 (LAMP-1) (Pharmingen, San Diego, CA), mouse monoclonal antibodies against human cathepsin D (R&D Systems, Minneapolis, MN), mouse monoclonal antibodies against human occludin (Invitrogen, Waltham, MA), and mouse monoclonal antibodies against human claudin-1 (Invitrogen, Waltham, MA). Cy3-conjugated F(ab′)_2_ fragments of goat anti-rabbit IgG and fluorescein isothiocyanate (FITC)-conjugated F(ab´)_2_ fragments of goat anti-rabbit IgG, (all from Jackson ImmunoResearch, West Grove, PA). FITC-conjugated F(ab´)_2_ fragments of goat anti-mouse IgG (from Southern Biotechnology, Birmingham, AL), Cy3-conjugated F(ab´)_2_ fragments of goat anti-mouse IgG (Molecular Probes, Eugene, OR). The polyclonal rabbit anti-*B*. *parapertussis* antiserum was obtained as described elsewhere [31].

### Cell culture and epithelial models

The 16HBE14o- human bronchial epithelial cell line, established from normal bronchial epithelium by transfection with the SV40 genome defective in the origin of replication, was kindly provided by Dr. Dieter Gruenert [32]. Cells were recovered from liquid nitrogen frozen stocks and maintained in minimal essential medium (MEM) (Gibco, Grand Island, NY) supplemented with 10% (v/v) of fetal bovine serum (FBS) (Gibco) in fibronectin (Sigma, Saint Louis, MO)/collagen (Sigma)-coated flasks (Nunc, Rochester, N.Y). Cells were maintained at 37°C in a humidified atmosphere with 5% CO_2_ and routinely subcultured at 1:6 split ratios by incubation with 0.05% (w/v) trypsin-EDTA (Gibco) for 10 min at 37°C. For infection assays, 16HBE14o- cells were seeded in different numbers to generate subconfluent or confluent monolayers of polarized cells. Cells seeded to confluency (5x10^5^ cells per well on 24 well plates) on fibronectin/collagen-coated coverslips were incubated for 1 day to generate non-polarized monolayers without tight junctions (1-day-old non-polarized confluent monolayer), or for 7 days to generate polarized monolayers with intact tight junctions (7-day-old polarized monolayer) [33]. In some experiments, the polarized monolayer was wounded using a sterile pipette tip prior to infection as previously reported [34]. To generate islands of polarized cells, 5x10^3^ cells were seeded on collagen coated coverslips and cultured for 7 days to generate islands with intact tight junctions as previously reported [35]. The presence of tight junctions was monitored both by occludin and claudin-1 staining. To this end, cells were fixed with 4% (w/v) paraformaldehyde, washed with phosphate-buffered saline (PBS) and incubated for 10 min at room temperature with PBS containing 50 mM NH_4_Cl. After two washing steps, cells were permeabilized by incubation with PBS containing 0.1% (v/v) Triton X-100 and 0.2% (w/v) Bovine Serum Albumin (BSA) (30 min at room temperature), and further incubated overnight with mouse anti-human occludin monoclonal antibodies or anti-human claudin-1 monoclonal antibodies in the presence of 0.1% (v/v) Triton X-100 (Sigma) and 0.2% (w/v) BSA (Sigma). After three washes with PBS containing 0.1% (v/v) Triton X-100 and 0.2% (w/v) BSA, the cells were incubated with Cy3-conjugated F(ab´)_2_ fragments of goat anti-mouse antibodies. Isotype controls were run in parallel. The cells were then washed, the coverslips were mounted with an anti-fading agent, and microscopic analyses were performed using a DMLB microscope coupled to a DC100 camera (Leica Microscopy Systems Ltd., Heerbrugg, Switzerland).

### Quantification of attached and internalized bacteria

Bacterial attachment to and internalization by epithelial cells were evaluated by double staining and fluorescence microscopy, as described before [11]. Briefly, cells were washed twice with sterile PBS and infected with *B*. *parapertussis* suspended in MEM plus 0.2% (w/v) BSA at a multiplicity of infection (MOI) of 20 or 200 bacteria per cell. The bacterial inoculum was quantified by colony forming units (CFU) counts. After 5 hours of incubation at 37°C with 5% CO2, non-adherent bacteria were removed by three washing steps prior to fixation with 4% (w/v) paraformaldehyde for 10 min. After fixation, cells were washed once with PBS and incubated for 10 min at room temperature with PBS containing 50 mM NH_4_Cl. The attached and internalized bacteria were discriminated using a two-step labeling procedure and fluorescence microscopy. Surface-bound bacteria were detected by incubation with polyclonal rabbit anti-*B*. *parapertussis* antiserum (30 min at 4°C), followed by incubation with FITC-conjugated F(ab´)_2_ fragments of goat anti-rabbit IgG (30 min at 4°C). To determine the number of intracellular bacteria, after two washing steps, the cells were permeabilized by incubation with PBS containing 0.1% (w/v) saponin and 0.2% (w/v) BSA (30 min at 25°C) and further incubated with rabbit anti-*B*. *parapertussis* antiserum in the presence of 0.1% (w/v) saponin and 0.2% (w/v) BSA (30 min at 25°C). After two washing steps, the cells were incubated with Cy3-conjugated goat F(ab´)_2_ fragments of anti-rabbit IgG (30 min at 25°C) in the presence of 0.1% (w/v) saponin and 0.2% (w/v) BSA. After washing with PBS containing 0.1% (w/v) saponin and 0.2% (w/v) BSA, some samples were incubated with 4′,6-diamidino-2-phenylindole (DAPI) (Sigma) for 10 min. The cells were mounted on microscope slides and analyzed by fluorescence microscopy using a DMLB microscope coupled to a DC100 camera (Leica Microscopy Systems Ltd., Heerbrugg, Switzerland). The number of extracellular and intracellular bacteria was evaluated by examining at least 200 cells.

### Intracellular survival

Polymyxin B protection assays were performed as previously described [11]. Briefly, 7-day-old polarized monolayers were infected with *B*. *parapertussis* suspended in MEM plus 0.2% (w/v) BSA at an MOI of 20, as described above. After 5 hours of incubation at 37°C with 5% CO_2_, non-adherent bacteria were removed by three washing steps prior to incubation with 100 μg/ml polymyxin B sulfate (Sigma) in MEM supplemented with 10% (v/v) FBS for 1 h to kill extracellular bacteria. It is important to note that this antibiotic cannot penetrate the mammalian cells. The antibiotic concentration was then reduced to 5 μg/ml until the end of the experiment. Intracellular survival of *B*. *parapertussis* was determined at 6, 24, and 48 h post-infection as follows. The cells were washed three times with PBS and incubated with trypsin-EDTA to detach the cells from the well. Next, the cells were pelleted and lysed with 0.1% (w/v) saponin in sterile water, and serial dilutions of the lysates were rapidly plated onto BGA plates to enumerate CFU. In parallel, the number of CFU in the cell culture supernatants was examined, and no viable bacteria were detected at any time post-infection.

In some experiments, after treatment with 100 μg/ml polymyxin B for 1 h and other 24 h in the presence of 5 μg/ml polymyxin B, the cells were washed to remove the antibiotic and further incubated with MEM supplemented with 10% (v/v) FBS, with or without the addition of polymyxin B (5 μg/mL), for 24 h. The presence of viable bacteria in the cell culture supernatants was then determined by CFU counts, as described above.

Control experiments were performed in parallel to assess the efficacy of the bactericidal activity of polymyxin B (100 μg/ml). Briefly, samples of 5x10^8^ bacteria were incubated with the antibiotic for 1 h at 37°C and plated on BGA. This resulted in a 99.999% decrease in CFU.

### Confocal microscopy analysis

Colocalization studies were performed as described before [11], with minor modifications. Briefly, the cells were incubated with *B*. *parapertussis* suspended in MEM plus 0.2% (w/v) BSA at an MOI of 20 as described above. After 5 hours of incubation at 37°C with 5% CO2, the cells were washed to remove non-attached bacteria and further incubated with 100 μg/ml polymyxin B in MEM supplemented with 10% (v/v) FBS for 1 h at 37°C to kill extracellular bacteria. Next, the concentration of polymyxin B was reduced to 5 μg/ml. At 6, 24, and 48 h post-infection, the cells were washed with PBS prior to fixation with 4% (w/v) paraformaldehyde and then incubated for 10 min at room temperature with PBS containing 50 mM NH_4_Cl. For bacterial colocalization with LAMP-1 or cathepsin D, the cells were washed, permeabilized by incubation with PBS containing 0.1% (w/v) saponin and 0.2% (w/v) BSA (30 min at room temperature), and incubated with either mouse anti-human LAMP-1 monoclonal antibodies plus rabbit anti-*B*. *parapertussis* serum (30 min at room temperature) or mouse anti-human cathepsin D monoclonal antibodies plus rabbit anti-*B*. *parapertussis* serum (30 min at room temperature) in the presence of 0.1% (w/v) saponin and 0.2% (w/v) BSA. After three washes with 0.1% (w/v) saponin and 0.2% (w/v) BSA, the cells were incubated with Cy3-conjugated F(ab′)_2_ fragments of goat anti-rabbit IgG and FITC-conjugated F(ab´)2 fragments of goat anti-mouse IgG for 30 min at room temperature in the presence of 0.1% (w/v) saponin and 0.2% (w/v) BSA. BSA. After washing with 0.1% (w/v) saponin and 0.2% (w/v) BSA, the coverslips were mounted on microscope slides and analyzed by confocal laser scanning microscope (TCS SP5; Leica). Isotype controls were run in parallel. The percentage of phagosomes containing bacteria that colocalized with a given marker was calculated by analyzing at least 50 phagosomes per sample.

For samples in which *B*. *parapertussis* and occludin colocalization was investigated the cells were washed twice, permeabilized by incubation with PBS containing 0.1% (v/v) Triton X-100 and 0.2% (w/v) BSA (30 min at room temperature), and incubated with mouse anti-human occludin monoclonal antibodies plus rabbit anti-*B*. *parapertussis* serum (overnight at 4°C) in the presence of 0.1% (v/v) Triton X-100 and 0.2% (w/v) BSA. After three washes with 0.1% (v/v) Triton X-100 and 0.2% (v/v) BSA, the cells were incubated with Cy3-conjugated F(ab´)_2_ fragments of goat anti-mouse IgG and FITC-conjugated F(ab´)_2_ fragments of goat anti-rabbit IgG for 30 min at room temperature in the presence of 0.1% (v/v) Triton X-100 and 0.2% (w/v) BSA. After washing with 0.1% (v/v) Triton X-100 and 0.2% (w/v) BSA, the coverslips were mounted on microscope slides and analyzed by confocal microscopy. Isotype controls were run in parallel.

For transferrin uptake assays, infection was performed as described before [11]. Forty-eight hours after infection, the cells were depleted of transferrin by three washes and subsequently incubated in MEM containing 1% (w/v) BSA for 10 min. The cells were then incubated for 30 min with Alexa594-Transferrin (Molecular probes) in an excess of BSA (1% (w/v)) to saturate non-specific binding. Finally, cells were fixed in 4% (w/v) paraformaldehyde. Cell-associated bacteria (both intra and intracellular bacteria) were stained as described above. After washing, the coverslips were mounted on microscope slides and analyzed by confocal microscopy. At least 50 phagosomes per sample were analyzed for colocalization with transferrin.

### Colocalization analysis

Colocalization analysis was performed using ImageJ (http://rsb.info.nih.gov/ij/) and the "view5D" plugin (https://nanoimaging.de/View5D/) for manual determination. The analysis was performed for each phagosome, generating an overlay chart that combined histogram information from the red (bacteria) and green (transferrin, LAMP-1, or cathepsin) channels. Positive colocalization was established when peaks in the red histogram overlapped with peaks in the green histogram. Colocalization were further validated using the "Colocalization threshold" plugin (https://imagej.net/plugins/colocalization-threshold).

### Tight junctions and monolayer integrity evaluation

Seven-day-old polarized monolayer grown in 24 well-plates were incubated with wild type *B*. *parapertussis* (MOI: 1), BppΔcyaA (MOI: 1), or medium alone in the absence of polymyxin B. The bacteria were then allowed to grow in the cell culture medium over the time of infection. The integrity of the occludin and claudin-1 network patterns were monitored by fluorescence microscopy at 6, 24, and 48 hours after infection, as described above. Isotype controls were run in parallel. Cells were washed, the coverslips were mounted with an anti-fading agent, and microscopic analyses were performed using a DMLB microscope coupled to a DC100 camera (Leica Microscopy Systems Ltd., Heerbrugg, Switzerland).

Additionally, the integrity of the monolayers incubated with medium alone, or infected with either the wild type *B*. *parapertussis*, or BppΔcyaA was evaluated as described previously [36], with minor modifications. To this end, 7-day-old polarized monolayer grown in 24 well-plates were incubated with wild type *B*. *parapertussis* (MOI 1), BppΔcyaA (MOI 1), or medium alone in the absence of polymyxin B. The bacteria were then allowed to grow in the cell culture medium over the time of infection. At 6, 24, and 48 h post-infection the detached cells were washed away with PBS and the remaining attached cells were fixed and stained with crystal violet. The integrity of the cell monolayer was evaluated by microscopy analysis as previously described [36], with minor modifications. Briefly, microscopy images of at least 3 random fields were captured with a 400X magnification and used to calculate the area that was deprived of cells by using the image processing software, ImageJ (National Institutes of Health, Bethesda, MD, http://imagej.nih.gov/ij/). The degree of disruption was calculated as the percentage of the total area deprived of cells and was calculated as follows. Degree of disruption = 100 x (total field area–area with cells)/(total field area).

### Statistical analysis

Student’s t test (95% confidence level) or analysis of variance (ANOVA) was used for statistical data evaluation. The significance of the differences between the mean values of the data evaluated by ANOVA was determined with the least-significant-difference (LSD) test at a 95% confidence level. Results are shown as mean ± standard deviation (SD).

## Results

### *B*. *parapertussis* preferentially attaches around the tight junctions of polarized epithelial cells

**Fig 1:**
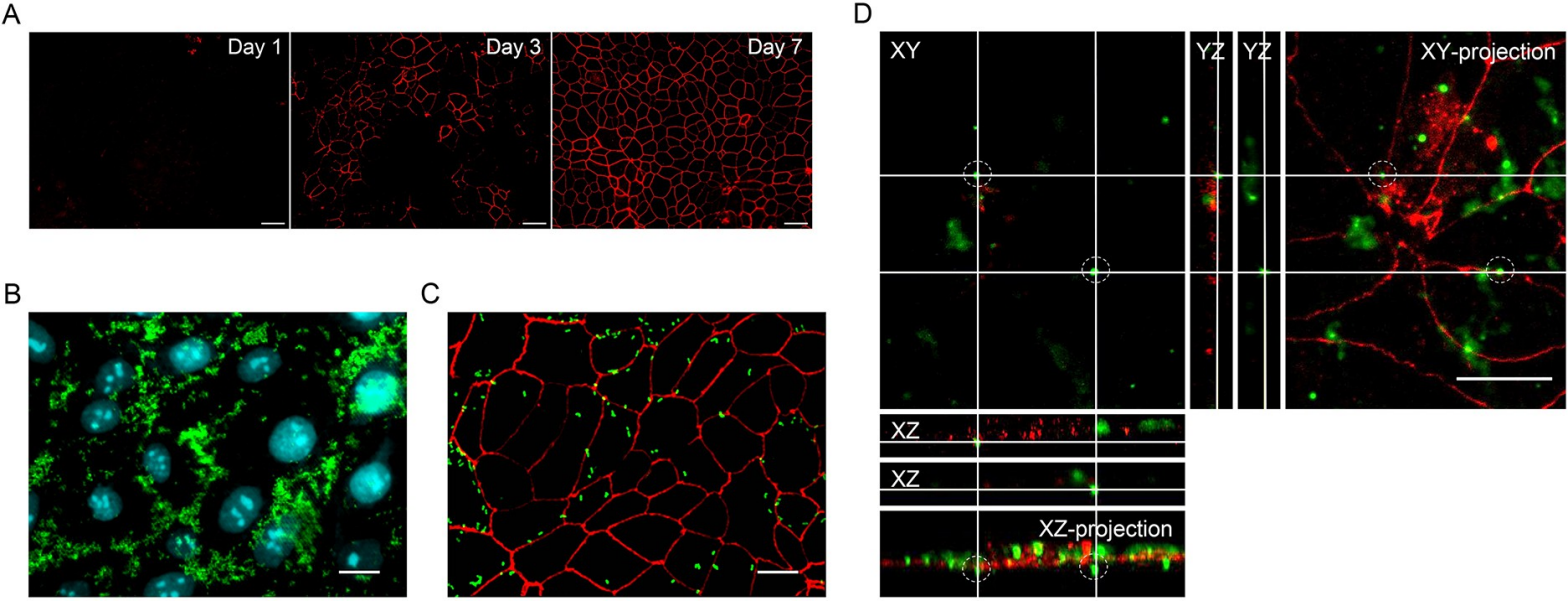
Interaction of *B*. *parapertussis* with polarized epithelial cells. (A) Representative fluorescence microscopy images of the occludin pattern of 16HBE14o- confluent monolayers at different times post-seeding. An occludin network-like pattern characteristic of intact tight junctions is observed 7 days after seeding. Scale bar: 20 μm. (B) Seven-day-old polarized monolayers were infected with *B*. *parapertussis* (MOI 20) for 5 h at 37°C, washed, fixed, and permeabilized before labeling the cell-associated bacteria with a green fluorescent dye. The nuclei were stained with DAPI (cyan). Representative fluorescence microscopy images are shown. Scale bar: 10 μm. (C) Seven-day-old polarized monolayers were infected with *B*. *parapertussis* (MOI 20) for 5 h at 37°C, washed, fixed, and permeabilized prior to labeling cell-associated bacteria (green) and tight junctions (red). Representative fluorescence microscopy images are shown. Scale bar: 10 μm. (D) Seven-day-old polarized monolayers infected with *B*. *parapertussis* (MOI 20) for 5 h at 37°C were washed, fixed, and permeabilized prior to labeling both cell-associated bacteria with a green fluorescent dye and tight junctions with a red fluorescent dye. Twenty confocal planes were acquired by confocal microscopy and processed using the ImageJ software to obtain a Z-stack. Orthogonal views (XZ and YZ) show two bacteria (dotted line) located in the monolayer middle sections. The XY- and XZ-projections (2D maximum intensity projection images obtained from a Z-stack of 20 confocal microscopy images using Imagej software) show that these bacteria are located in a confocal plane below the tight junction. Representative confocal microscopy images are shown. Scale bar: 10 μm.

In order to gain some insight into the interaction of *B*. *parapertussis* with the respiratory epithelium during infection, we used a confluent monolayer of polarized 16HBE14o- cells, in which intact tight junctions were formed. To obtain polarized monolayers, 16HBE14o- cells were seeded at confluent density onto 24-well plates containing cover slips. The development of functional tight junctions was checked by fluorescence microscopy imaging of immune-stained protein occludin at different days post-seeding. Fig 1A shows that the occludin pattern characteristic of closed tight junctions was obtained on day 7 post-seeding. The interaction of *B*. *parapertussis* with polarized epithelial cells was then evaluated in this model. To this end, *B*. *parapertussis* was incubated with the 7-day-old polarized monolayer of 16HBE14o- cells at an MOI of 200 for 5 h at 37°C. After washing to remove non-adherent bacteria, cell-associated bacteria were immunostained and imaged by fluorescence microscopy. Fig 1B shows bacteria mainly attached close to the intercellular boundaries of polarized cells, suggesting a bacterial tropism for these sites. To better dissect this issue, we reduced the multiplicity of infection to 20 bacteria per cell. Five hours after infection, the cells were washed to remove non-adherent bacteria, and both cell-associated bacteria and tight junctions were immunostained and imaged by fluorescence microscopy. Fig 1C shows bacteria preferentially attached around the tight junctions, suggesting that these structures are preferred sites of attachment for *B*. *parapertussis*. The analysis of XY confocal planes together with XY- and XZ-projections showed several bacteria located below the tight junction marker occludin (Fig 1D), suggesting that the interaction with the tight junctions might allow *B*. *parapertussis* to access the intercellular space of the polarized monolayers.

### *B*. *parapertussis* disrupts the tight junctions in an adenylate cyclase dependent way

**Fig 2:**
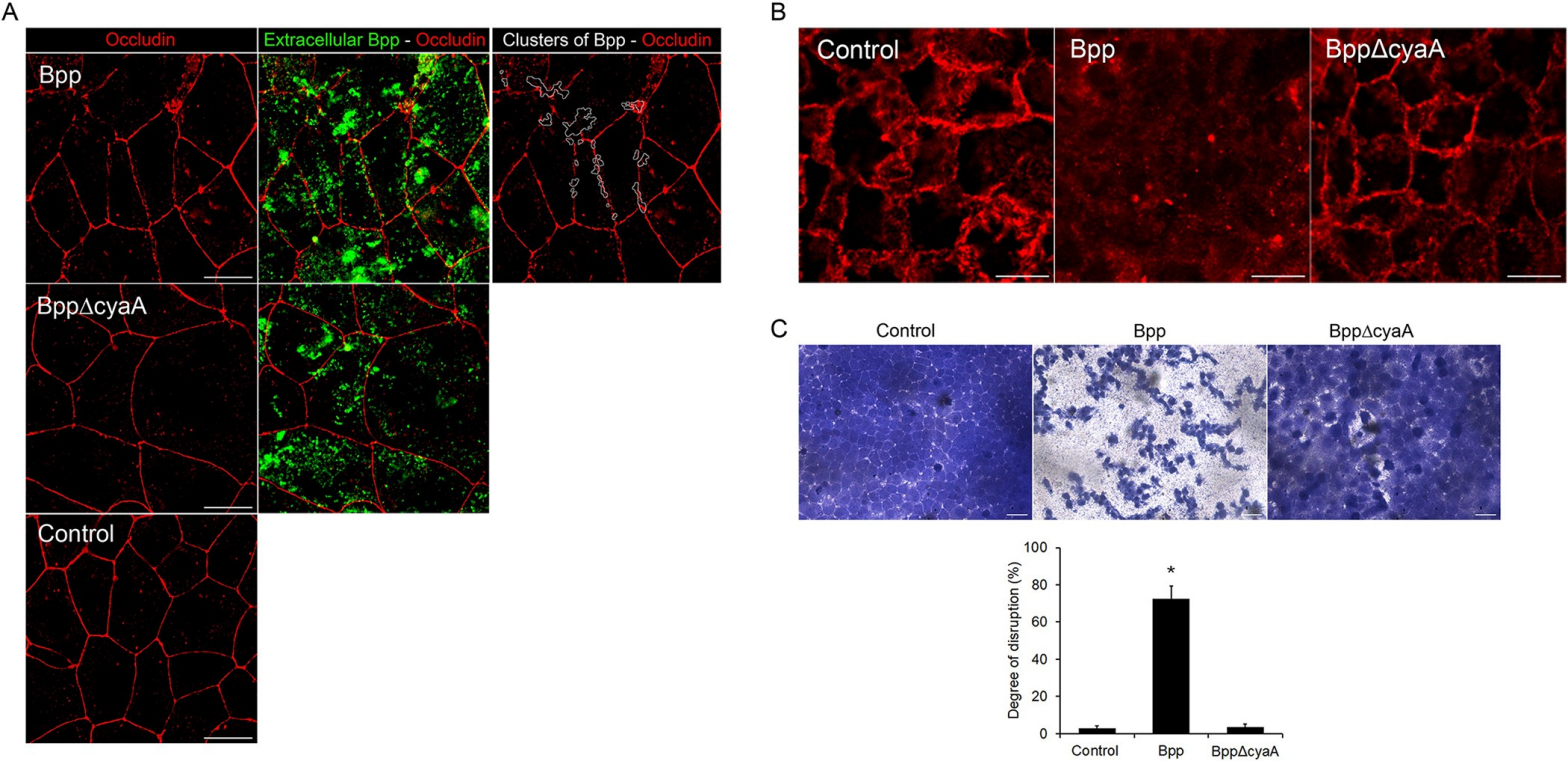
The CyaA toxin of *B*. *parapertussis* promotes the disruption of tight junctions and affects the integrity of polarized monolayers. (A) Seven-day-old polarized monolayers were incubated with wild type *B*. *parapertussis* (Bpp) (MOI 1), a CyaA deficient mutant (BppΔCyaA) (MOI 1), or medium alone (control) for 6, 24 and 48 h at 37°C, washed, fixed and permeabilized prior to labeling tight junctions with A) mouse anti-human occludin antibodies or B) mouse anti-human claudin-1 antibodies followed by Cy3-conjugated F(ab´)2 fragments of goat anti-mouse IgG, and both extracellular and intracellular bacteria with a green fluorescent dye. Representative fluorescence microscopy images at 24 h post-infection are shown. Scale bar: 10 μm. Clusters of wild type *B*. *parapertussis* at the site where tight junctions are disrupted 24 h post-infection are shown at a higher magnification. C) Seven-day-old polarized monolayers were incubated with wild type *B*. *parapertussis* (Bpp) (MOI 1), a CyaA defficient mutant (BppΔCyaA) (MOI 1), or medium alone (control) for 6, 24 and 48 h at 37°C. The monolayers were washed to remove the detached cells and fixed prior to labeling with cristal violet. Representative fluorescence microscopy images 48 h post-infection are shown. Scale bar: 20 μm. The bars represent the mean ± SD of three independent experiments, each performed in triplicate. The bars show the mean degree of disruption of the epithelial monolayers, calculated as the percentage of the total area deprived of cells. The degree of disruption found in monolayers infected with the wild-type bacteria was significantly higher than the degree of disruption found in monolayers incubated with the *B*. *parapertussis* CyaA-deficient mutant or medium alone (*P <0,001).

We next investigated whether *B*. *parapertussis* is able to disrupt tight junctions, which would eventually expose the epithelial intercellular space and allow the bacteria access to this site. To this end, 7-day-old polarized monolayers were infected with *B*. *parapertussis* (MOI 1) in the absence of polymyxin B to allow bacterial infection to proceed. At 6, 24, and 48 h after infection cell-associated bacteria and tight junctions were stained and analyzed by fluorescence microscopy. The occludin network pattern was found disrupted 24 hours post-infection (Fig 2A). In order to assess whether the CyaA of *B*. *parapertussis* was involved in the disruption of tight junctions as previously found for *B*. *pertussis* CyaA [37], we infected a 7-day-old polarized monolayer with an isogenic CyaA deficient mutant of *B*. *parapertussis* (MOI 1) in the absence of polymyxin B. Fig 2A shows that, unlike *B*. *parapertussis* wild type, the infection with the CyaA deficient mutant or medium alone (control) did not affect the integrity of the tight junctions 24 h post-infection. Fig 2A further shows that the disruption of occludin network was mainly visible in areas in which wild type *B*. *parapertussis* was found in clusters (S1 Fig), suggesting that the disruption of tight junctions might depend on the local concentration of CyaA toxin. A second marker, claudin-1, was used to evaluate TJ integrity. Fig 2B shows that 24 hours post-infection Bpp promotes a CyaA-dependent delocalization of claudin-1. In parallel, the integrity of the monolayer was further evaluated by crystal violet staining as described in [36]. Fig 2C shows that 48 hours after infection, *B*. *parapertussis* wild type, but not the CyaA deficient mutant strain, significantly disrupted the cell monolayer integrity, confirming the role of *B*. *parapertussis* CyaA in the process.

### *B*. *parapertussis* adenylate cyclase plays a role in the bacterial access to the intracellular space of polarized epithelial cells

**Fig 3:**
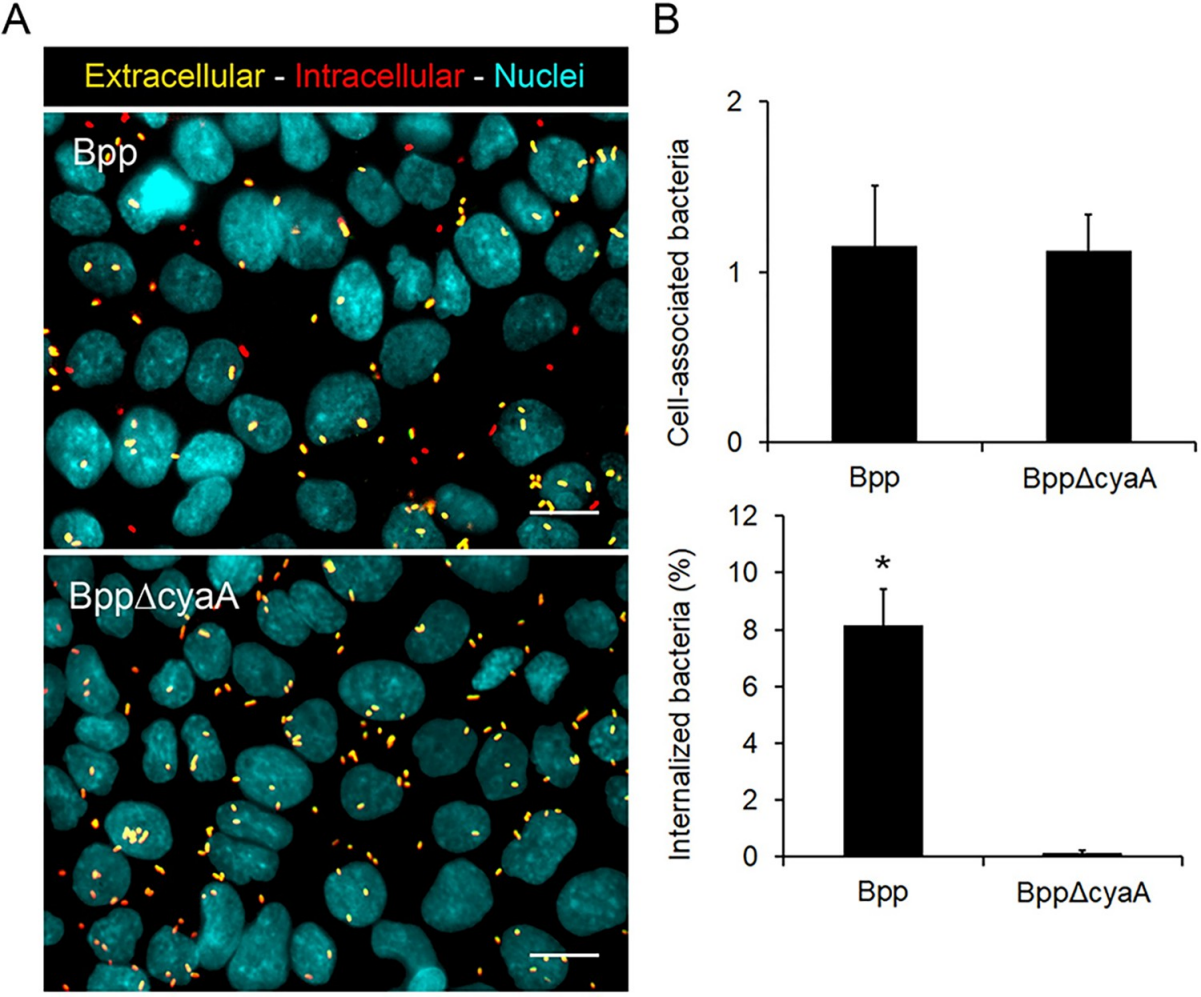
The CyaA toxin promotes access of *B*. *parapertussis* to the intracellular space of epithelial cells in polarized monolayers. (A) Seven-day-old polarized monolayers were infected (MOI 20) with *B*. *parapertussis* wild type strain (Bpp) or a CyaA deficient mutant (BppΔCya) for 5 h at 37°C, washed to remove non-attached bacteria, fixed, and permeabilized prior to labeling the intracellular bacteria with red fluorescent dye and extracellular bacteria with both green and red fluorescent dyes. The nuclei were stained with DAPI (cyan). Representative florescence microscopy images are shown. Scale bar: 10 μm. (B) Cell-associated and internalized *B*. *parapertussis* were determined by analyzing fluorescence microscopy images. A total of 200 cells per condition were analyzed. Cell-associated bacteria were defined as the number of extracellular and intracellular bacteria per cell. Internalization was expressed as the percentage of the cell-associated bacteria that were found inside the cell. Bars represent the means ± SD of the results of two independent experiments. The percentage of internalized wild type bacteria was significantly higher than the percentage of internalized *B*. *parapertussis* CyaA deficient mutant (*P<0.05).

In order to investigate whether tight junctions disruption by the CyaA toxin of *B*. *parapertussis* promotes the bacterial access to the intracellular space of epithelial cells, we compared the infection efficiency of the wild type and the CyaA deficient mutant of *B*. *parapertussis* on a 7-day-old polarized monolayer. To this end, the polarized monolayer was infected with either the wild type strain or an isogenic CyaA deficient mutant of *B*. *parapertussis* (MOI 20) for 5 h at 37°C. Non-attached extracellular bacteria were removed by washing, and both intracellular and extracellular bacteria were enumerated by double staining and fluorescence microscopy. Fig 3 shows that the attachment levels (assessed as the number of cell-associated bacteria) of both strains were similar. However, the percentage of internalized CyaA deficient mutant strain was significantly lower than the percentage of internalized wild type bacteria (Fig 3), suggesting that the activity of CyaA toxin promotes the access of *B*. *parapertussis* to the intracellular space of polarized monolayers.

### *B*. *parapertussis* attachment and internalization are more efficient in cells exposing the components of the basolateral membrane

**Fig 4:**
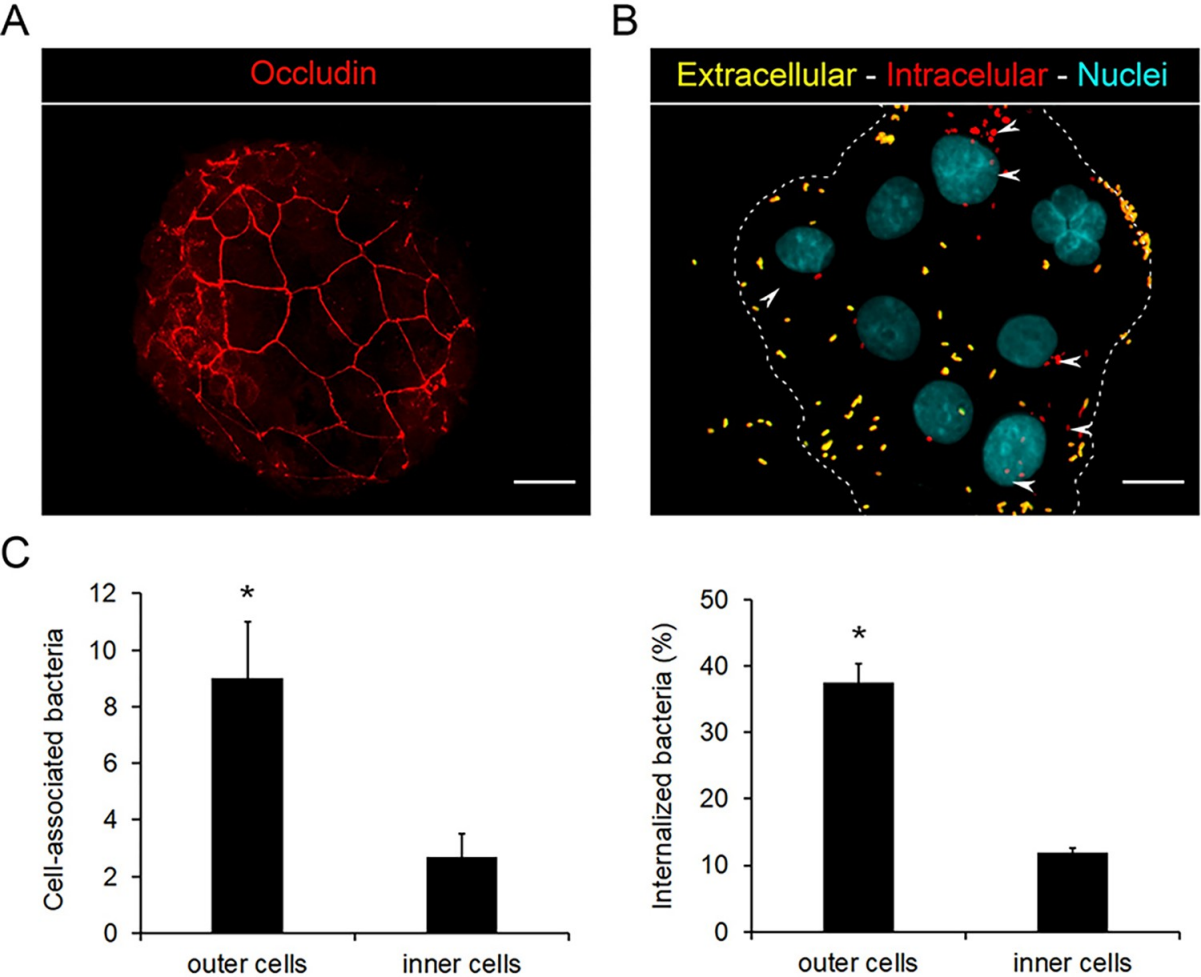
*B*. *parapertussis* preferentially attaches to the basolateral membrane of epithelial cells and gets access to the intracellular location preferentially through this membrane. (A) Representative fluorescence microscopy images showing the occludin staining pattern of islands of 7-day-old polarized cells incubated at 37°C and 5% CO2 for 7 days. The occludin network-like pattern, characteristic of functional tight junctions, is observed at the center of the island and not at the edges of the island, where components of the basolateral membranes are exposed. Scale bar: 10 μm (B) Polarized islands (7-day-old) were infected with *B*. *parapertussis* (MOI 20) for 5 h at 37°C. Next, the cells were washed to remove non-attached bacteria, fixed, and permeabilized prior to labeling the intracellular bacteria with a red fluorescent dye and extracellular bacteria with both green and red fluorescent dyes. Representative florescence microscopy images are shown. Scale bar: 10 μm. Intracellular bacteria are indicated by white arrowheads. (C) Cell-associated and internalized bacteria were determined by fluorescence microscopy. At least 200 cells were analyzed per experiment. The number of cell-associated bacteria (both extracellular and intracellular) in either the outer or inner cells of the island is shown. Internalization was expressed as the percentage of cell-associated bacteria found intracellularly. The mean ± SD of three independent experiments is shown. The number of cell-associated bacteria was significantly higher in the outer cells than in the inner cells of the island (*P<0.05). The percentage of internalized bacteria was significantly higher in the outer cells than in the inner cells (*P<0.05).

We next investigated whether, as found for other pathogens that disrupt tight junctions [34], *B*. *parapertussis* preferentially targets the components of the basolateral membrane. To this end, we used a subconfluent model in which 16HBE14o- cells were allowed to grow into a small group of polarized cells (herein called islands). In this model the tight junctions separate the basolateral and apical membrane components of the cells located in the center of the island (inner cells). Therefore, these cells solely expose the apical membrane, while cells located at the edge of the island (outer cells) also exposed the components of basolateral membranes [34, 35]. The development of tight junctions in the islands was confirmed by occludin staining at 7 days post-seeding (Fig 4A). The islands were then infected with *B*. *parapertussis* (MOI 20) for 5 h at 37°C. Surface-attached and intracellular bacteria were discriminated by double-staining as described above. Fig 4C shows that the number of cell-associated bacteria (both extracellular and intracellular) to the outer cells was significantly higher than the number of bacteria associated to the inner cells suggesting that *B*. *parapertussis* preferentially attaches to cells exposing components of the basolateral membrane. The percentage of internalized bacteria was also higher in the cells located at the edge of the islands than in the inner cells. Five hours after infection 92,9 ± 1,6% of intracellular bacteria were found in outer cells suggesting that the bacteria get more efficiently internalized in cells exposing the components of the basolateral membrane.

### Wounded edges of polarized monolayers are docking sites for *B*. *parapertussis* attachment and internalization

**Fig 5:**
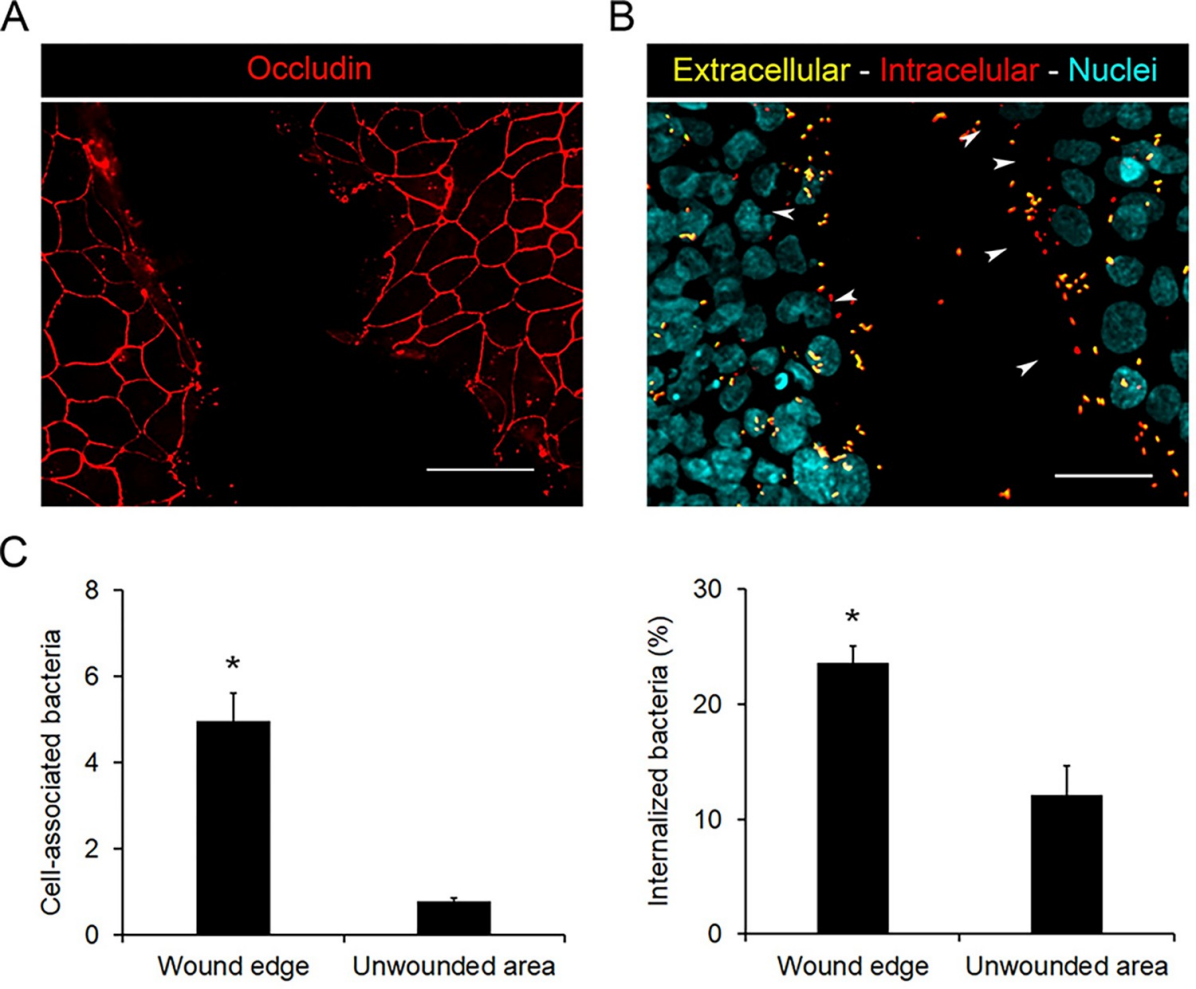
Injured areas of the polarized monolayers are docking zones for *B*. *parapertussis* attachment and internalization. (A) Representative fluorescence microscopy images showing the occluding pattern of an artificially wounded polarized monolayer. Scale bar: 20 μm (B) Intact 7-day-old polarized monolayers were wounded using a sterile pipette tip and infected with *B*. *parapertussis* (MOI 20) for 5 h at 37°C. Next, the cells were washed to remove non-attached bacteria, fixed, and permeabilized prior to labeling the intracellular bacteria with a red fluorescent dye and the extracellular bacteria with both green and red fluorescent dyes. The nuclei were stained with DAPI (cyan). Representative florescence microscopy images are shown. Scale bar: 20 μm. Intracellular bacteria (red) are indicated by white arrowheads. (C) Cell-associated (both intra and extracellular) and internalized bacteria were determined by analyzing fluorescence microscopy images. At least 200 cells per experiment were analyzed. Cell-associated bacteria were expressed as the number of bacteria (both extracellular and intracellular bacteria) per cell located either at the wounded edges or unwounded areas of the monolayer. The bacterial internalization was expressed as the percentage of the cell-associated bacteria that were located intracellularly. The mean ± SD of two independent experiments are shown. Both the number of cell-associated bacteria and the percentage of internalized bacteria were significantly higher in cells located at the wound edges than in cells located in unwounded areas of the monolayer (*P<0.05).

We next evaluated the interaction of *B*. *parapertussis* with a wounded polarized monolayer that is expected to expose basolateral proteins at the wound edge [34]. To this end, a 7-day-old polarized monolayer was artificially wounded using a sterile pipette tip and infected with *B*. *parapertussis* (MOI 20). Five hours post-infection the number of cell-associated bacteria was determined both at the wound edges and in the unwounded area by fluorescence microscopy as described above. As can been seen in Fig 5, a higher number of bacteria were attached to the wound edges as compared with the unwounded areas, suggesting that *B*. *parapertussis* attached more efficiently to areas eventually exposing the components of the basolateral membranes. Accordingly, the percentage of internalized bacteria in the cells located at the wound edges was significantly higher than those found in cells of unwounded areas (24,2 ± 2% and 8,6 ± 1%, respectively).

### Tight junctions restrict *B*. *parapertussis* attachment and entry into epithelial cells

**Fig 6:**
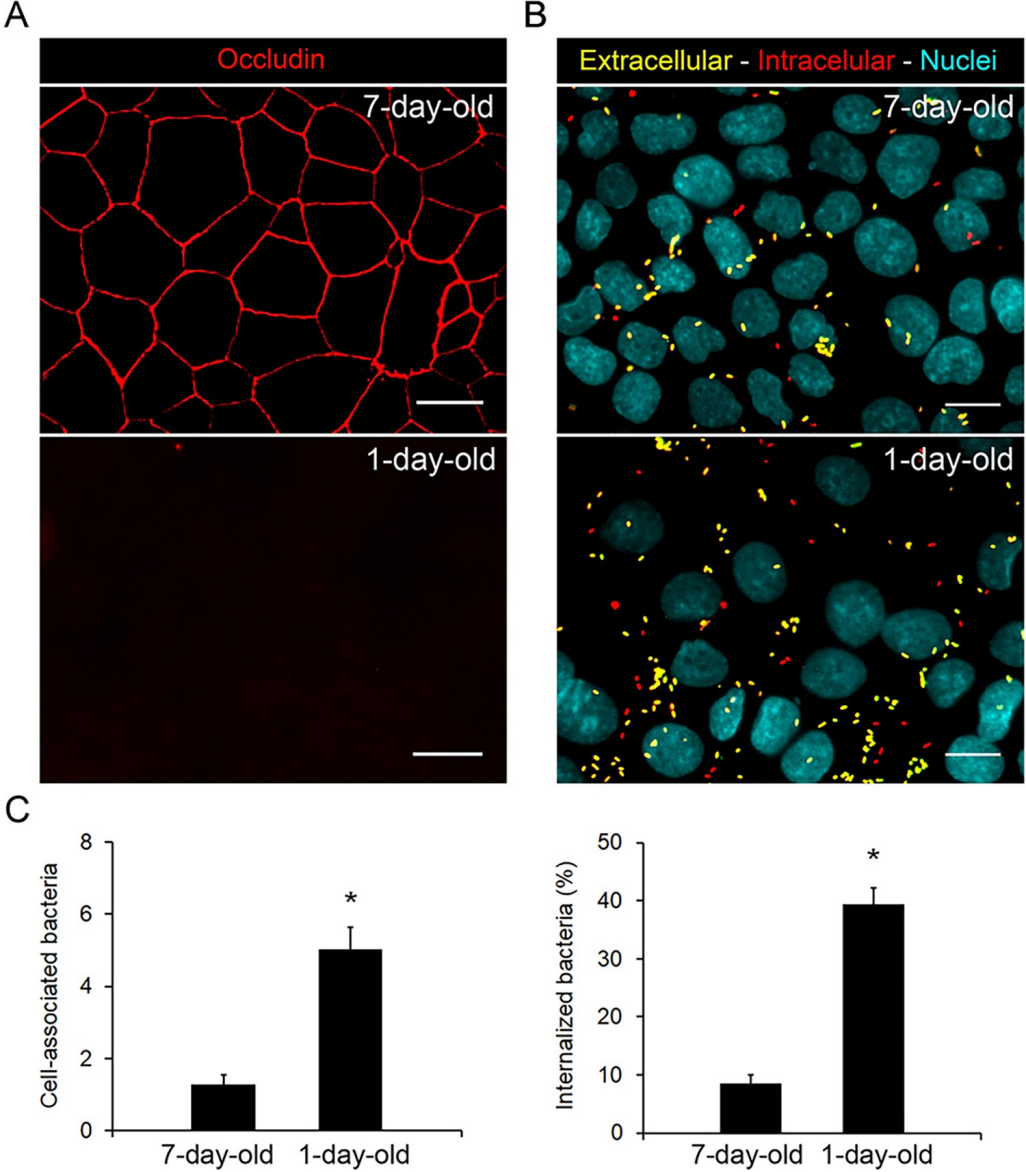
Influence of tight junctions on *B*. *parapertussis* attachment and entry into epithelial cells. (A) Seven-day-old and 1-day-old confluent monolayers were fixed and stained against occludin (red). The occludin network-like pattern characteristics of intact tight junctions is seen only in the polarized model. Representative fluorescence microscopy images are shown. Scale bar: 10 μm. (B) Seven-day-old and 1-day-old confluent monolayers were infected with *B*. *parapertussis* (MOI 20) for 5 h at 37°C, washed to remove non-attached bacteria, fixed, and permeabilized prior to labeling the intracellular bacteria with a red fluorescent dye and extracellular bacteria with both green and red fluorescent dyes. The nuclei were stained with DAPI (Cyan). Representative florescence microscopy images are shown. Scale bar: 10 μm. (C) Extracellular and intracellular *B*. *parapertussis* were determined using fluorescence microscopy. At least 200 cells per condition were analyzed. The number of cell-associated bacteria (both extracellular and intracellular) is shown. Bacterial internalization was expressed as the percentage of cell-associated bacteria found intracellularly. The mean ± SD of two independent experiments, each performed in quadruplicate, are shown. The number of both cell-associated and internalized bacteria in 7-day-old polarized cells was significantly different from those in 1-day-old monolayer cells (*P<0.05).

Finally, we compared *B*. *parapertussis* attachment and internalization by a 7-day-old polarized monolayer and a 1-day-old non-polarized confluent monolayer lacking tight junctions. In the 1-day-old non-polarized confluent monolayer docking molecules belonging to the basolateral membrane, such as heparan sulfate proteoglycans, lipid rafts, and α5β1 integrin [18, 34, 38–42], among others, are expected to be exposed. We observed that the attachment and internalization of *B*. *parapertussis* was significantly higher in the non-polarized confluent monolayer than in the polarized monolayer (Fig 6). As observed in previous studies [34], these results might indicate the exposure of target sites of the basolateral membrane in the non-polarized monolayer, which are unavailable for bacterial binding in the polarized model.

Taken together, these results suggest that the basolateral membrane represents a better docking site for *B*. *parapertussis* as compared with the apical membrane, and that the tight junctions restrict bacterial access to this preferred target and eventually to the intracellular location.

### *B*. *parapertussis* remains alive inside the polarized epithelial cells

**Fig 7:**
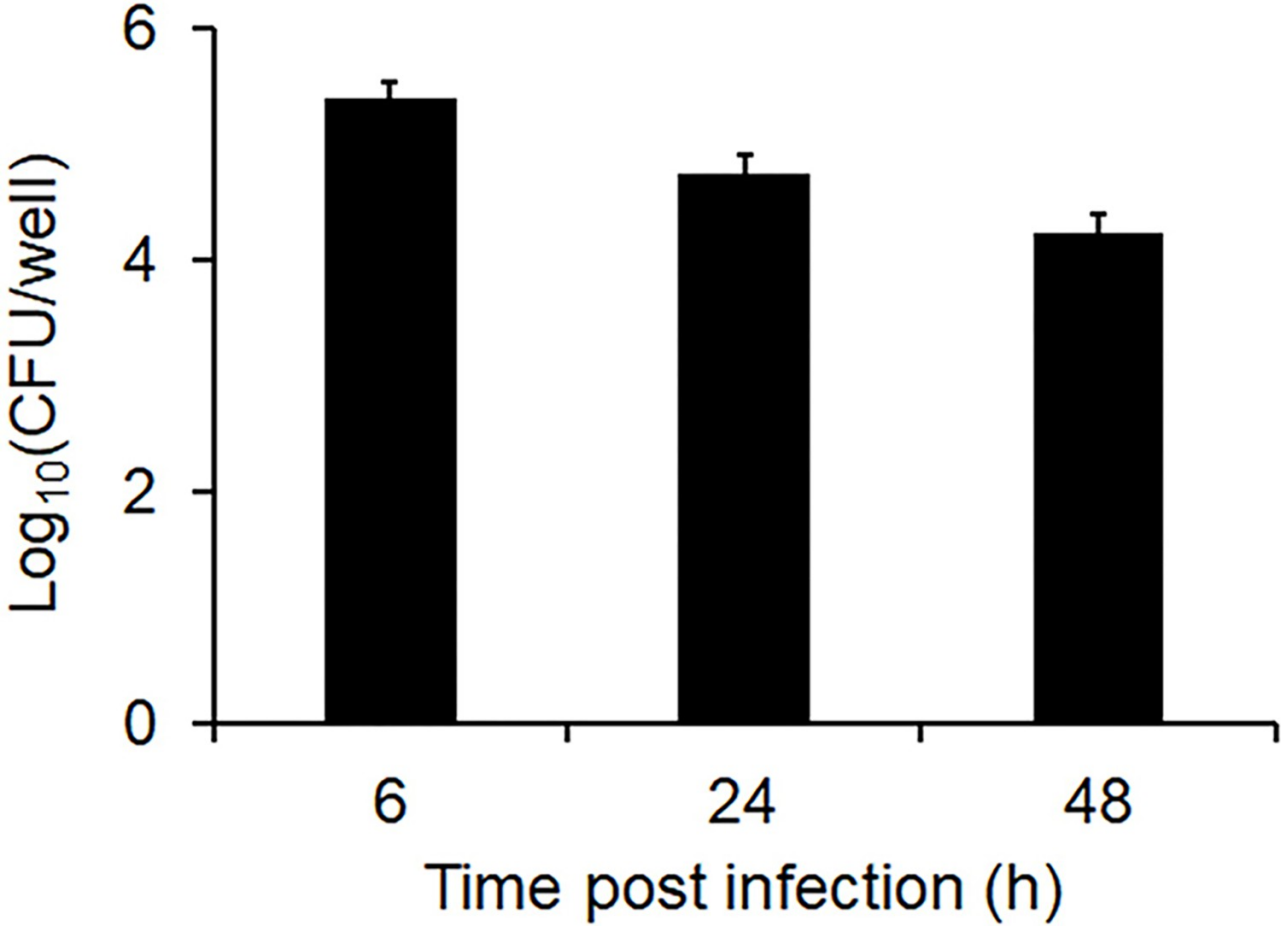
Intracellular survival of *B*. *parapertussis* in epithelial polarized cells. Seven-day-old polarized monolayers were infected with *B*. *parapertussis* (MOI 20) for 5 h at 37°C, washed to remove non-attached bacteria, and treated with polymyxin B (100 μg/ml). After 1 h at 37°C, cells were washed and incubated in the presence of polymyxin B (5 μg/ml). At 6, 24, and 48 h post-infection, the cells were washed, lysed, and the number of viable intracellular bacteria was evaluated by CFU counts. The data represent the mean ± SD of three independent experiments.

We next analyzed the intracellular survival of *B*. *parapertussis* within polarized epithelial cells. To this end, 7-day-old monolayers were infected with *B*. *parapertussis* (MOI 20) for 5 h at 37°C. Non-attached extracellular bacteria were removed by washing and the cells were further incubated with a bactericidal concentration of Polymyxin B (100 μg/ml) for 1 h to kill extracellular bacteria. The cells were then washed and further incubated in the presence of a bacteriostatic concentration of Polymyxin B (5 μg/ml). At 6, 24, or 48 h post-infection, the cells were washed, lysed, and the number of viable intracellular bacteria determined by CFU counts. Fig 7 shows a high number of viable intracellular bacteria 6 h after infection, which did not significantly decrease over time post-infection, indicating that *B*. *parapertussis* remained alive within these cells.

### *B*. *parapertussis* precludes phagolysosomal fusion and has access to extracellular nutrients

**Fig 8:**
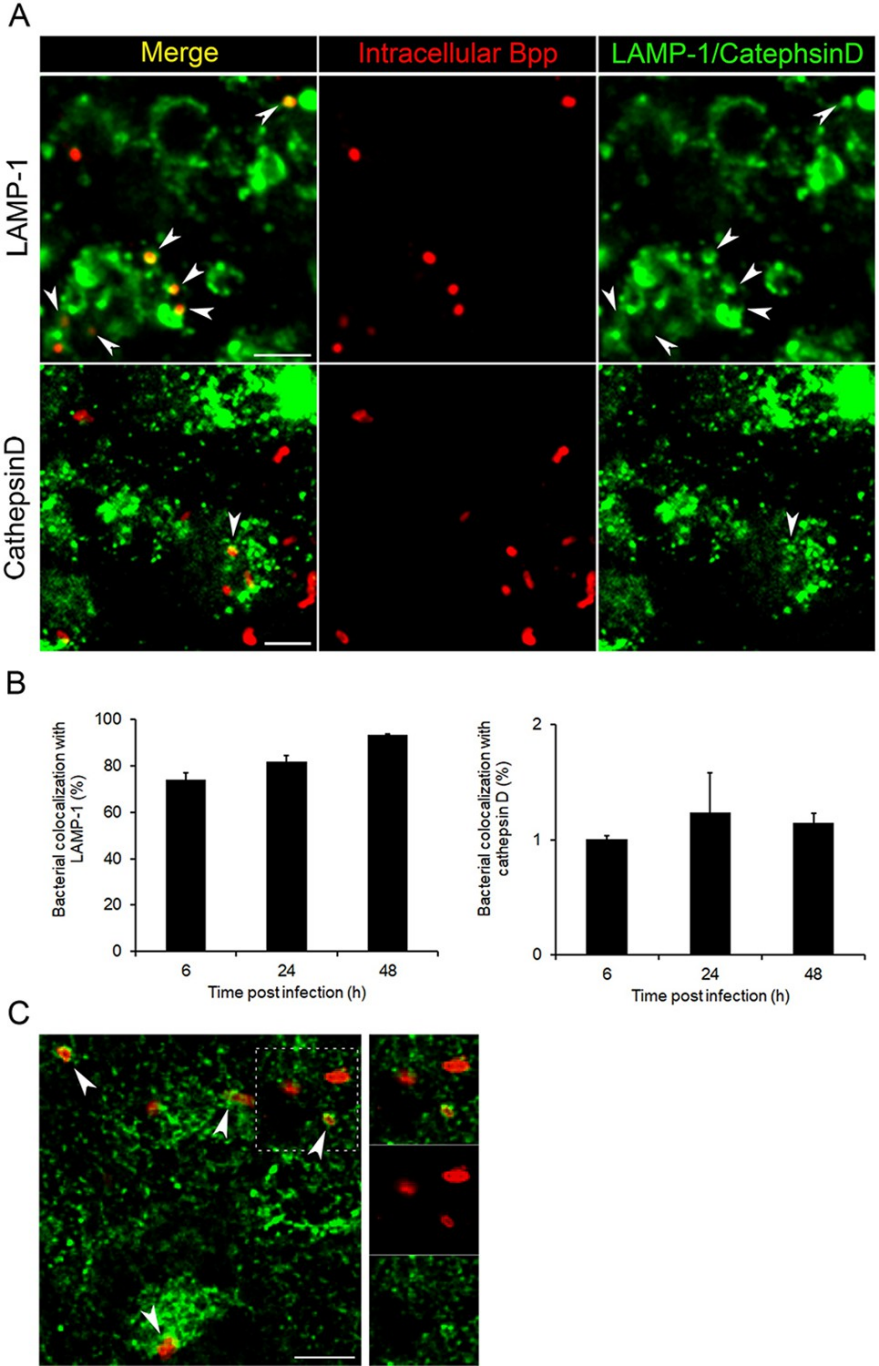
Intracellular trafficking of *B*. *parapertussis*. Seven-day-old polarized monolayers were infected with *B*. *parapertussis* (MOI 20) for 5 h at 37°C, washed to remove non-attached bacteria, treated with polymyxin B (100 μg/ml) for 1 h at 37°C, washed, and incubated at 37°C in the presence of polymyxin B (5 μg/ml). At different time points post–infection (6, 24, and 48 h), the cells were fixed and permeabilized prior to labeling the intracellular bacteria with a red fluorescent dye and (A) LAMP-1 (green) or (B) cathepsin D (green). Representative confocal images are shown. Scale bar: 5 μm. The colocalization of *B*. *parapertussis* with each marker is reflected by a red fluorescent *B*. *parapertussis* surrounded by green-labeled LAMP-1 or cathepsin D. The percentage of bacterial colocalization with each marker is shown. The bars represent the mean ± SD of two independent experiments. C) In selected experiments, 48 h after infection the cells were incubated with Alexa Fluor 594-transferrin (green) and fixed prior to labeling the bacteria with a red fluorescent dye. Representative confocal images are shown. Scale bar: 5 μm. The colocalization of *B*. *parapertussis* with transferrin is reflected by the red fluorescent *B*. *parapertussis* surrounded by green transferrin.

In order to gain some insight into the bacterial intracellular fate within polarized epithelial cells, we next investigated the trafficking of *B*. *parapertussis* in polarized monolayers. To this end, 7-day-old polarized monolayers were infected with *B*. *parapertussis* (MOI 20) for 5 h at 37°C. Non-attached extracellular bacteria were removed by washing and the cells were further incubated with a bactericidal concentration of Polymyxin B for 1 h to kill extracellular bacteria. The cells were then washed and further incubated in the presence of a bacteriostatic concentration of Polymyxin B. At 6, 24, or 48 h points post-infection bacterial colocalization with late endosomal (LAMP-1) and lysosomal (cathepsin D) markers was investigated. Fig 8 shows a high percentage of bacteria colocalizing with LAMP-1 early after infection that remained unchanged over time post-infection. On the other hand, the percentage of bacterial colocalization with the lysosomal marker cathepsin D was low from the beginning of the infection and did not change over the time of infection. These results seem to indicate that phagosomes containing *B*. *parapertussis* quickly acquired late endosomal markers, but in agreement with the high intracellular survival observed in Fig 7, their maturation into lysosomes was mostly precluded. Accordingly, in control experiments in which infected cells were pulsed with Alexa Fluor 594-transferrin (a recycling pathway marker) and analyzed by confocal microscopy, intracellular *B*. *parapertussis* was found colocalizing with exogenous transferrin 48 h after infection (Fig 8). These results indicate that the intracellular bacteria reside in phagosomes that fuse with recycling vesicles, gaining access to nutrients coming from the extracellular space.

### Intracellular *B*. *parapertussis* is able to repopulate the extracellular environment

**Fig 9:**
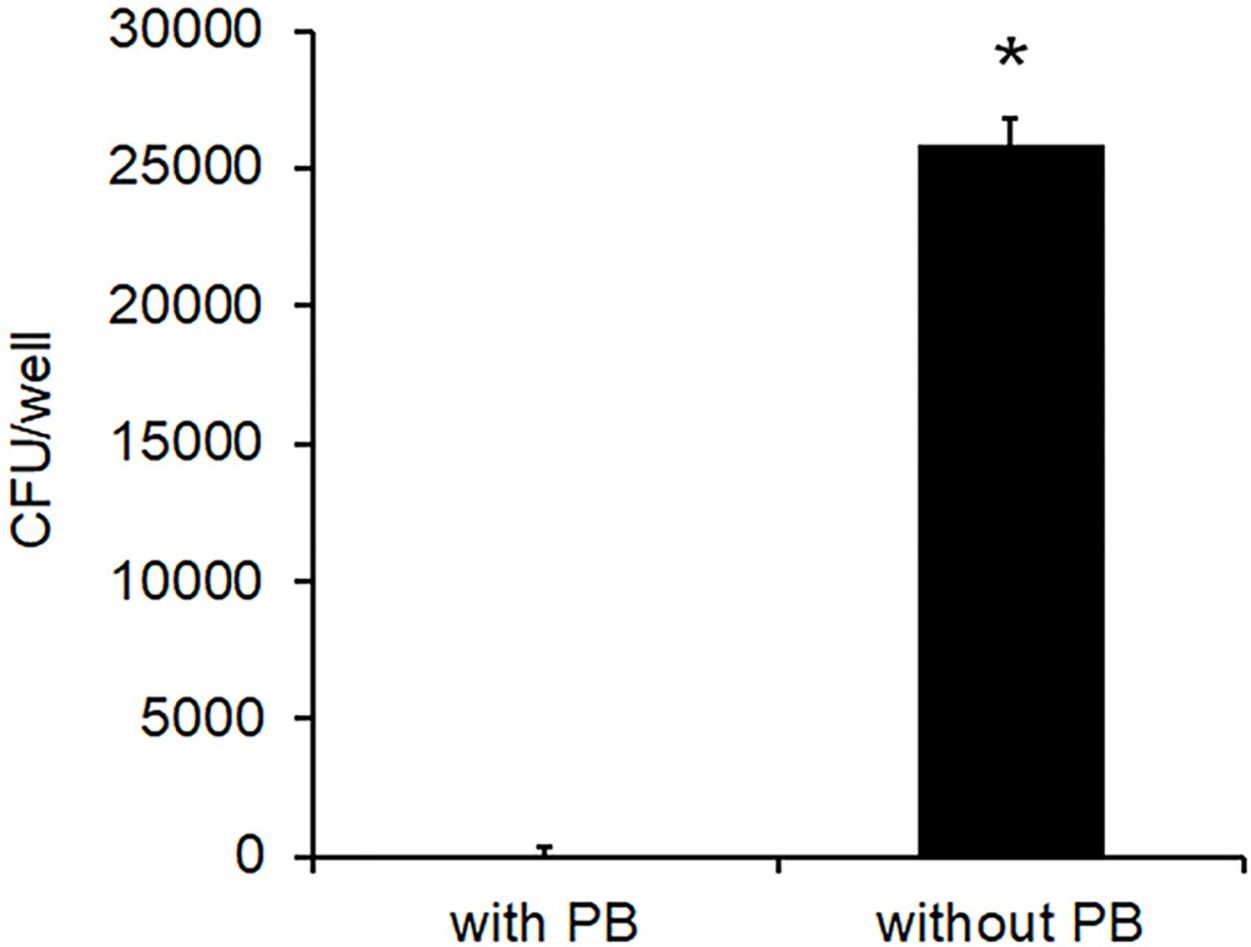
The intracellular *B*. *parapertussis* can repopulate extracellular medium. Seven-day-old polarized monolayers were infected with *B*. *parapertussis* (MOI 20) for 5 h at 37°C, washed to remove non-attached bacteria treated with polymyxin B (100 μg/ml) for 1 h at 37°C. After washing, cells were further incubated for 24 h in the presence of polymyxin B (5 μg/ml). One sample was washed and incubated with fresh antibiotic-free medium, and the other sample was maintained in medium containing polymyxin B (5 μg/ml). After 24 h, the number of viable bacteria in the extracellular medium was determined by CFU counts. The data represent the mean ± SD of three independent experiments. The number of viable bacteria in the extracellular medium was significantly higher in the absence of antibiotic than in its presence (*P<0.05).

Finally, we evaluated whether intracellular *B*. *parapertussis* is able to reach alive the extracellular environment. To this end, 7-day-old polarized monolayers were incubated with *B*. *parapertussis* (MOI 20) for 5 h at 37°C. Non-attached extracellular bacteria were removed by washing and the cells were further incubated with 100 μg/ml Polymyxin B for 1 h to kill extracellular bacteria, washed, and incubated in the presence of bacteriostatic concentration of 5 μg/ml of polymyxin B for 24 h. The cells were then washed again, and further incubated in the presence or absence of polymyxin B (5 μg/ml) for another 24 h. At this time point, the number of bacteria in the supernatant of these two samples was evaluated by counting the colony forming units. The number of bacteria in the supernatant of cells incubated in the absence of antibiotic significantly increased over the time of infection (Fig 9), indicating that intracellular viable bacteria can repopulate the extracellular medium.

## Discussion

*B*. *parapertussis* is transmitted from host to host within vaccinated populations, causing either classic illness or asymptomatic/mild infections. Whereas classic pertussis patients can be identified and treated, mild and asymptomatic patients generally represent an unnoticed carrier that eventually contribute to the dissemination of this pathogen [5, 43–45]. Since this is an obligate human pathogen, better knowledge of bacterial persistence within the host might contribute to the development of better preventive strategies.

We have previously found that *B*. *parapertussis* remains alive inside human professional phagocytes within phagosomes that do not undergo lysosomal maturation [11], suggesting that *B*. *parapertussis* might have an intracellular stage during the infectious process. Here, we found evidence indicating that epithelial cells of the respiratory tract have the potential to be a niche of persistence for this pathogen.

The airway epithelium constitutes a physical barrier against pathogens and its function depends on cell polarity and tight junctions [16]. In this study, we used a polarized model of cells with a healthy pulmonary origin, which retains differentiated epithelial morphology and function [32], and demonstrated to be a good surrogate for human airway epithelium [46]. When seeded on fibronectin/collagen coated surfaces, these cells form a monolayer of polarized cells with tight junctions that segregate apical and basolateral membrane components [33].

Using this model, we observed that *B*. *parapertussis* attaches next or on top of the intercellular boundaries of polarized epithelial cells, and disrupts the tight junctions in a CyaA-dependent manner gaining access to the basolateral membrane, through which it gets efficiently internalized by the cell.

The attachment pattern of *B*. *parapertussis* to polarized monolayers might be driven by the bacterial affinity for tight junctions-associated proteins, as previously found for other pathogens [16, 23], and/or to the high concentration of lipid rafts [19, 38–41], known docking platforms for *B*. *parapertussis*, [11, 12, 52], in these structures. The attachment to tight junctions might play a key role in the opening of these structures by enabling the local accumulation of CyaA in their proximity. This last assumption seems supported by the fact that tight junctions were found disrupted mainly in areas where *B*. *parapertussis* was found in clusters. By disrupting the tight junction the bacteria get access to the intercellular space (Fig 1D) where it might deliver CyaA directly into the surroundings of the basolateral membrane. Since previous studies have shown a higher impact of adenylate cyclase toxin if it is delivered through the basolateral membrane as compared to the apical membrane [47], the bacterial tropism for the tight junctions would also facilitate CyaA delivery at a site where it is most effective eventually further promoting the tight junctions opening.

Previous studies have demonstrated that CyaA of *B*. *pertussis* can disrupt cellular tight junctions [37]. While previous reports have suggested that eukaryotic host factor 14-3-3, expressed by eukaryotic cells, selectively inactivates the CyaA toxin expressed by *B*. *parapertussis* [48], our recently published study showed that *B*. *parapertussis* CyaA is active in macrophages [30]. In this study, we demonstrate that *B*. *parapertussis* CyaA is also active enough to compromise the tight junction integrity and barrier function of the epithelial monolayer. In experiments in which the bacterial growth was not restricted by antibiotics the infection by *B*. *parapertussis* compromised the tight junction integrity of the polarized monolayer and the cells began to detach from the monolayer in a CyaA-dependent way as the infection progressed. Since in these studies the growth of *B*. *parapertussis* was not restricted the observed disruption of the tight junctions and cell detachment could be due to the intoxication of cells by the increase in the number of bacteria and/or in the level of different bacterial toxins in the culture medium. However, since neither the disruption of tight junctions nor cell detachment was observed when the infection was performed with a CyaA deficient mutant, these effects can be attributed exclusively to this toxin. The disruption of tight junctions by CyaA of *B*. *parapertussis* is consistent with the reported CyaA-mediated activation of host calpains, a calcium-dependent cysteine protease that cleaves tight junctions proteins, occludin, among others [49]. Previous studies have shown that CyaA of *B*. *pertussis* leads to a cAMP-dependent activation of PKA, which activates calcium channels with L-type properties [50], promoting a calcium influx that eventually activates the host calpains [51].

Taken together, our results suggest that *B*. *parapertussis* gains access to preferred docking molecules located at the basolateral membrane by disrupting tight junctions through the action of CyaA. Moreover, cell detachment induced by the bacterium could generate a gap in the epithelial monolayer, which can eventually be exploited to reach the basolateral components. The bacterial higher affinity and internalization by cells exposing components of the basolateral membrane suggests a differential concentration of docking molecules or different proteins in this membrane compared to the apical membrane. Although this issue requires further investigation, the basolateral membrane enrichment in lipid rafts [19, 38–41], previously found to be involved in *B*. *parapertussis* attachment to host cells [11, 12, 52], and in α5β1 integrin, which was found to be involved in *Bordetella* internalization by epithelial cells [42], might play a role in the observed process.

By granting access to the basolateral membrane components of polarized epithelial cells, *B*. *parapertussis* CyaA facilitates bacterial entry into these cells, where the bacteria survive for days in phagosomes that do not undergo lysosomal maturation, as determined by the lack of Cathepsin D in most phagosomes two days after infection. The low colocalization of internalized *B*. *parapertussis* with cathepsin D, a protease that accumulates in lysosomes, indicates that the majority of intracellular bacteria do not reach mature lysosomes, but remain in alternative intracellular compartments. This type of LAMP+/Cathepsin D- phagosome was previously associated with a niche of persistence for other pathogens such as *Helicobacter pylori* [53], and *Escherichia coli* [54] in epithelial cells. In agreement with the high intracellular survival rate over the time of infection, we found that intracellular *B*. *parapertussis* has access to nutrients from the recycling pathway, as determined by bacterial colocalization with transferrin. Confirming bacterial intracellular viability, *B*. *parapertussis* was found able to repopulate the extracellular medium, suggesting that this pathogen might be able to develop a niche of persistence within these cells.

Summarizing, although *B*. *parapertussis* is regarded as a non-invasive pathogen whose proliferation is restricted to the respiratory mucosal surfaces, our results show that this pathogen might be able to hijack the barrier function of the respiratory epithelium in a CyaA dependent manner, gaining access to the components of the basolateral membrane through which it can develop an intracellular infection that might be the basis of bacterial persistence within the host. Additional studies are needed to further unveil whether the ability of *B*. *parapertussis* to disrupt the epithelial barrier enables bacterial dissemination to the underlying tissues.

## Supporting information

S1 Fig Occludin pattern disruption at sites where *B*. *parapertussis* forms clusters. Seven-day-old polarized monolayers incubated with wild type *B*. *parapertussis* (Bpp) (MOI 1), a CyaA deficient mutant (BppΔCyaA) (MOI 1), or medium alone (control) for 24 h at 37°C, were washed, fixed and permeabilized prior to labeling tight junctions protein occludin (red) and cell-associated bacteria (green). Fluorescence microscopy images were taked at 24 hours post-infection and analyzed using the ImageJ plugin "view 5D" to generate overlay charts from the sum intensity x- and y-projection, combining histogram information from the red (occludin) and green (cell-associated bacteria) channels at specific xy points. Scale bar: 10 μm.
(TIF)

S1 File Raw data of Fig 2C. (XLSX)

S2 File Raw data of Fig 3. (XLSX)

S3 File Raw data of Fig 4. (XLSX)

S4 File Raw data of Fig 5. (XLSX)

S5 File Raw data of Fig 6. (XLSX)

S6 File Raw data of Fig 7. (XLSX)

S7 File Raw data of Fig 8. (XLSX)

S8 File Raw data of Fig 9. (XLSX)

## References

[1] Pinell-McNamara VA, Acosta AM, Pedreira MC, Carvalho AF, Pawloski L, Tondella ML, et al. Expanding Pertussis Epidemiology in 6 Latin America Countries through the Latin American Pertussis Project. Emerg Infect Dis. 2017;23(13). doi: 10.3201/eid2313.170457 29155677 PMC5711316

[2] Cherry JD, Seaton BL. Patterns of *Bordetella parapertussis* Respiratory Illnesses: 2008–2010. Clin Infect Dis. 2012;54(4):534–7. 22156857 10.1093/cid/cir860

[3] Bokhari H, Said F, Syed MA, Mughal A, Kazi YF, Heuvelman K, et al. Whooping cough in Pakistan: *Bordetella pertussis* vs *Bordetella parapertussis* in 2005–2009. Scand J Infect Dis. 2011;43(10):818–20. 21563881 10.3109/00365548.2011.577804

[4] Watanabe M, Nagai M. Whooping cough due to Bordetella parapertussis: an unresolved problem. Expert Rev Anti Infect Ther. 2004;2(3):447–54. doi: 10.1586/14787210.2.3.447 15482209

[5] Liese JG, Renner C, Stojanov S, Belohradsky BH. Clinical and epidemiological picture of *B*. *pertussis* and *B*. *parapertussis* infections after introduction of acellular pertussis vaccines. Arch Dis Child. 2003;88(8):684–7. 12876162 10.1136/adc.88.8.684 PMC1719607

[6] Kurova N, Njamkepo E, Brun D, Tseneva G, Guiso N. Monitoring of Bordetella isolates circulating in Saint Petersburg, Russia between 2001 and 2009. Res Microbiol. 2010;161(10):810–5. doi: 10.1016/j.resmic.2010.09.013 20870020

[7] David S, van Furth R, Mooi FR. Efficacies of whole cell and acellular pertussis vaccines against *Bordetella parapertussis* in a mouse model. Vaccine. 2004;22(15–16):1892–8. 15121300 10.1016/j.vaccine.2003.11.005

[8] Zhang X, Rodriguez ME, Harvill ET. O antigen allows *B*. *parapertussis* to evade *B*. *pertussis* vaccine-induced immunity by blocking binding and functions of cross-reactive antibodies. PLoS One. 2009;4(9):e6989. doi: 10.1371/journal.pone.0006989 19750010 PMC2737124

[9] Khelef N, Danve B, Quentin-Millet MJ, Guiso N. *Bordetella pertussis* and *Bordetella parapertussis*: two immunologically distinct species. Infect Immun. 1993;61(2):486–90. doi: 10.1128/iai.61.2.486–490.1993 8423077 PMC302754

[10] Watanabe M, Nagai M. Reciprocal protective immunity against *Bordetella pertussis* and *Bordetella parapertussis* in a murine model of respiratory infection. Infect Immun. 2001;69(11):6981–6. 11598073 10.1128/IAI.69.11.6981-6986.2001 PMC100078

[11] Gorgojo J, Harvill ET, Rodriguez ME. *Bordetella parapertussis* Survives inside Human Macrophages in Lipid Raft-Enriched Phagosomes. Infect Immun. 2014;82(12):5175–84. doi: 10.1128/IAI.02553-14 25267839 PMC4249269

[12] Gorgojo J, Lamberti Y, Valdez H, Harvill ET, Rodriguez ME. *Bordetella parapertussis* survives the innate interaction with human neutrophils by impairing bactericidal trafficking inside the cell through a lipid raft-dependent mechanism mediated by the lipopolysaccharide O antigen. Infect Immun. 2012;80(12):4309–16. 23027528 10.1128/IAI.00662-12 PMC3497435

[13] Wolfe DN, Goebel EM, Bjornstad ON, Restif O, Harvill ET. The O antigen enables *Bordetella parapertussis* to avoid *Bordetella pertussis*-induced immunity. Infect Immun. 2007;75(10):4972–9. 17698566 10.1128/IAI.00763-07 PMC2044517

[14] Wolfe DN, Kirimanjeswara GS, Harvill ET. Clearance of *Bordetella parapertussis* from the lower respiratory tract requires humoral and cellular immunity. Infect Immun. 2005;73(10):6508–13. 16177324 10.1128/IAI.73.10.6508-6513.2005 PMC1230969

[15] Bertuzzi M, Hayes GE, Bignell EM. Microbial uptake by the respiratory epithelium: outcomes for host and pathogen. FEMS Microbiol Rev. 2019;43(2):145–61. doi: 10.1093/femsre/fuy045 30657899 PMC6435450

[16] Lu RY, Yang WX, Hu YJ. The role of epithelial tight junctions involved in pathogen infections. Mol Biol Rep. 2014;41(10):6591–610. doi: 10.1007/s11033-014-3543-5 24965148

[17] Ikenouchi J. Roles of membrane lipids in the organization of epithelial cells: Old and new problems. Tissue Barriers. 2018;6(2):1–8. 10.1080/21688370.2018.1502531 PMC6179127 30156967

[18] Bucior I, Mostov K, Engel JN. *Pseudomonas aeruginosa*-mediated damage requires distinct receptors at the apical and basolateral surfaces of the polarized epithelium. Infect Immun. 2010;78(3):939–53. 20008530 10.1128/IAI.01215-09 PMC2825949

[19] Hatayama S, Shimohata T, Amano S, Kido J, Nguyen AQ, Sato Y, et al. Cellular Tight Junctions Prevent Effective *Campylobacter jejuni* Invasion and Inflammatory Barrier Disruption Promoting Bacterial Invasion from Lateral Membrane in Polarized Intestinal Epithelial Cells. Front Cell Infect Microbiol. 2018;8:15. 29441328 10.3389/fcimb.2018.00015 PMC5797580

[20] Rose R, Hauser S, Stump-Guthier C, Weiss C, Rohde M, Kim KS, et al. Virulence factor-dependent basolateral invasion of choroid plexus epithelial cells by pathogenic *Escherichia coli* in vitro. FEMS Microbiol Lett. 2018;365(24). 10.1093/femsle/fny274 PMC7190931 30476042

[21] Finlay BB, Falkow S. Common themes in microbial pathogenicity revisited. Microbiol Mol Biol Rev. 1997;61(2):136–69. doi: 10.1128/mmbr.61.2.136-169.1997 9184008 PMC232605

[22] De Gaetano GV, Lentini G, Galbo R, Coppolino F, Fama A, Teti G, et al. Invasion and trafficking of hypervirulent group B streptococci in polarized enterocytes. PLoS One. 2021;16(6):e0253242. doi: 10.1371/journal.pone.0253242 34129624 PMC8205152

[23] Guttman JA, Finlay BB. Tight junctions as targets of infectious agents. Biochim Biophys Acta. 2009;1788(4):832–41. doi: 10.1016/j.bbamem.2008.10.028 19059200

[24] Chen ML, Ge Z, Fox JG, Schauer DB. Disruption of tight junctions and induction of proinflammatory cytokine responses in colonic epithelial cells by *Campylobacter jejuni*. Infect Immun. 2006;74(12):6581–9. 17015453 10.1128/IAI.00958-06 PMC1698078

[25] Simonovic I, Rosenberg J, Koutsouris A, Hecht G. Enteropathogenic *Escherichia coli* dephosphorylates and dissociates occludin from intestinal epithelial tight junctions. Cell Microbiol. 2000;2(4):305–15. 11207587 10.1046/j.1462-5822.2000.00055.x

[26] Perdomo JJ, Gounon P, Sansonetti PJ. Polymorphonuclear leukocyte transmigration promotes invasion of colonic epithelial monolayer by *Shigella flexneri*. J Clin Invest. 1994;93(2):633–43. 7906696 10.1172/JCI117015 PMC293886

[27] Golovkine G, Faudry E, Bouillot S, Elsen S, Attree I, Huber P. *Pseudomonas aeruginosa* Transmigrates at Epithelial Cell-Cell Junctions, Exploiting Sites of Cell Division and Senescent Cell Extrusion. PLoS Pathog. 2016;12(1):e1005377. 26727615 10.1371/journal.ppat.1005377 PMC4699652

[28] Pentecost M, Otto G, Theriot JA, Amieva MR. *Listeria monocytogenes* invades the epithelial junctions at sites of cell extrusion. PLoS Pathog. 2006;2(1):e3. 16446782 10.1371/journal.ppat.0020003 PMC1354196

[29] Allen A, Maskell D. The identification, cloning and mutagenesis of a genetic locus required for lipopolysaccharide biosynthesis in *Bordetella pertussis*. Mol Microbiol. 1996;19(1):37–52. 8821935 10.1046/j.1365-2958.1996.354877.x

[30] Carrica MDC, Gorgojo JP, Lamberti YA, Valdez HA, Rodriguez ME. *Bordetella parapertussis* adenylate cyclase toxin promotes the bacterial survival to the encounter with macrophages. Microb Pathog. 2023;174:105898. 36460144 10.1016/j.micpath.2022.105898

[31] Hellwig SM, van Oirschot HF, Hazenbos WL, van Spriel AB, Mooi FR, van De Winkel JG. Targeting to Fcgamma receptors, but not CR3 (CD11b/CD18), increases clearance of *Bordetella pertussis*. J Infect Dis. 2001;183(6):871–9. 11237803 10.1086/319266

[32] Cozens AL, Yezzi MJ, Kunzelmann K, Ohrui T, Chin L, Eng K, et al. CFTR expression and chloride secretion in polarized immortal human bronchial epithelial cells. Am J Respir Cell Mol Biol. 1994;10(1):38–47. doi: 10.1165/ajrcmb.10.1.7507342 7507342

[33] Grumbach Y, Quynh NV, Chiron R, Urbach V. LXA4 stimulates ZO-1 expression and transepithelial electrical resistance in human airway epithelial (16HBE14o-) cells. Am J Physiol Lung Cell Mol Physiol. 2009;296(1):L101–8. doi: 10.1152/ajplung.00018.2008 18849442

[34] Pedersen GA, Jensen HH, Schelde AB, Toft C, Pedersen HN, Ulrichsen M, et al. The basolateral vesicle sorting machinery and basolateral proteins are recruited to the site of enteropathogenic *E*. *coli* microcolony growth at the apical membrane. PLoS One. 2017;12(6):e0179122. 28636623 10.1371/journal.pone.0179122 PMC5479554

[35] Bouwman LI, Niewold P, van Putten JP. Basolateral invasion and trafficking of *Campylobacter jejuni* in polarized epithelial cells. PLoS One. 2013;8(1):e54759. doi: 10.1371/journal.pone.0054759 23382959 PMC3557275

[36] Landoni VI, Schierloh P, de Campos Nebel M, Fernandez GC, Calatayud C, Lapponi MJ, et al. Shiga toxin 1 induces on lipopolysaccharide-treated astrocytes the release of tumor necrosis factor-alpha that alter brain-like endothelium integrity. PLoS Pathog. 2012;8(3):e1002632. doi: 10.1371/journal.ppat.1002632 22479186 PMC3315494

[37] Hasan S, Kulkarni NN, Asbjarnarson A, Linhartova I, Osicka R, Sebo P, et al. *Bordetella pertussis* Adenylate Cyclase Toxin Disrupts Functional Integrity of Bronchial Epithelial Layers. Infect Immun. 2018;86(3). 10.1128/IAI.00445-17 PMC5820963 29203545

[38] Head BP, Patel HH, Insel PA. Interaction of membrane/lipid rafts with the cytoskeleton: impact on signaling and function: membrane/lipid rafts, mediators of cytoskeletal arrangement and cell signaling. Biochim Biophys Acta. 2014;1838(2):532–45. doi: 10.1016/j.bbamem.2013.07.018 23899502 PMC3867519

[39] Li Q, Zhang Q, Wang C, Li N, Li J. Invasion of enteropathogenic Escherichia coli into host cells through epithelial tight junctions. FEBS J. 2008;275(23):6022–32. doi: 10.1111/j.1742-4658.2008.06731.x 19016848

[40] Stamatovic SM, Johnson AM, Sladojevic N, Keep RF, Andjelkovic AV. Endocytosis of tight junction proteins and the regulation of degradation and recycling. Ann N Y Acad Sci. 2017;1397(1):54–65. doi: 10.1111/nyas.13346 28415156 PMC5479724

[41] Nusrat A, Parkos CA, Verkade P, Foley CS, Liang TW, Innis-Whitehouse W, et al. Tight junctions are membrane microdomains. J Cell Sci. 2000;113 (Pt 10):1771–81. doi: 10.1242/jcs.113.10.1771 10769208

[42] Ishibashi Y, Relman DA, Nishikawa A. Invasion of human respiratory epithelial cells by *Bordetella pertussis*: possible role for a filamentous hemagglutinin Arg-Gly-Asp sequence and alpha5beta1 integrin. Microb Pathog. 2001;30(5):279–88. 11373122 10.1006/mpat.2001.0432

[43] Mattoo S, Cherry JD. Molecular pathogenesis, epidemiology, and clinical manifestations of respiratory infections due to *Bordetella pertussis* and other Bordetella subspecies. Clin Microbiol Rev. 2005;18(2):326–82. 15831828 10.1128/CMR.18.2.326-382.2005 PMC1082800

[44] He Q, Viljanen MK, Arvilommi H, Aittanen B, Mertsola J. Whooping cough caused by *Bordetella pertussis* and *Bordetella parapertussis* in an immunized population. JAMA. 1998;280(7):635–7. 9718056 10.1001/jama.280.7.635

[45] Zhang Q, Yin Z, Li Y, Luo H, Shao Z, Gao Y, et al. Prevalence of asymptomatic *Bordetella pertussis* and *Bordetella parapertussis* infections among school children in China as determined by pooled real-time PCR: a cross-sectional study. Scand J Infect Dis. 2014;46(4):280–7. 24520981 10.3109/00365548.2013.878034

[46] Gangl K, Reininger R, Bernhard D, Campana R, Pree I, Reisinger J, et al. Cigarette smoke facilitates allergen penetration across respiratory epithelium. Allergy. 2009;64(3):398–405. doi: 10.1111/j.1398-9995.2008.01861.x 19120070

[47] Eby JC, Ciesla WP, Hamman W, Donato GM, Pickles RJ, Hewlett EL, et al. Selective translocation of the *Bordetella pertussis* adenylate cyclase toxin across the basolateral membranes of polarized epithelial cells. J Biol Chem. 2010;285(14):10662–70. 20139088 10.1074/jbc.M109.089219 PMC2856274

[48] Fukui-Miyazaki A, Toshima H, Hiramatsu Y, Okada K, Nakamura K, Ishigaki K, et al. The Eukaryotic Host Factor 14-3-3 Inactivates Adenylate Cyclase Toxins of *Bordetella bronchiseptica* and *B*. *parapertussis*, but Not *B*. *pertussis*. mBio. 2018;9(4). 10.1128/mBio.00628-18 PMC6113625 30154257

[49] Chun J, Prince A. Ca2+ signaling in airway epithelial cells facilitates leukocyte recruitment and transepithelial migration. J Leukoc Biol. 2009;86(5):1135–44. doi: 10.1189/jlb.0209072 19605699 PMC3192023

[50] Martin C, Gomez-Bilbao G, Ostolaza H. Bordetella adenylate cyclase toxin promotes calcium entry into both CD11b+ and CD11b- cells through cAMP-dependent L-type-like calcium channels. J Biol Chem. 2010;285(1):357–64. doi: 10.1074/jbc.M109.003491 19875442 PMC2804183

[51] Uribe KB, Etxebarria A, Martin C, Ostolaza H. Calpain-Mediated Processing of Adenylate Cyclase Toxin Generates a Cytosolic Soluble Catalytically Active N-Terminal Domain. PLoS One. 2013;8(6):e67648. doi: 10.1371/journal.pone.0067648 23840759 PMC3694075

[52] Gorgojo J, Scharrig E, Gomez RM, Harvill ET, Rodriguez ME. *Bordetella parapertussis* Circumvents Neutrophil Extracellular Bactericidal Mechanisms. PLoS One. 2017;12(1):e0169936. doi: 10.1371/journal.pone.0169936 28095485 PMC5240980

[53] Terebiznik MR, Vazquez CL, Torbicki K, Banks D, Wang T, Hong W, et al. *Helicobacter pylori* VacA toxin promotes bacterial intracellular survival in gastric epithelial cells. Infect Immun. 2006;74(12):6599–614. 17000720 10.1128/IAI.01085-06 PMC1698066

[54] Mysorekar IU, Hultgren SJ. Mechanisms of uropathogenic *Escherichia coli* persistence and eradication from the urinary tract. Proc Natl Acad Sci U S A. 2006;103(38):14170–5. 16968784 10.1073/pnas.0602136103 PMC1564066

