# Supplementary figures and images for: Adenylate cyclase toxin of Bordetella parapertussis disrupts the epithelial barrier granting the bacterial access to the intracellular space of epithelial cells
Source: PLoS One. 2023 Nov 27;18(11):e0291331. doi: 10.1371/journal.pone.0291331 (PMC10681170; doi:10.1371/journal.pone.0291331)

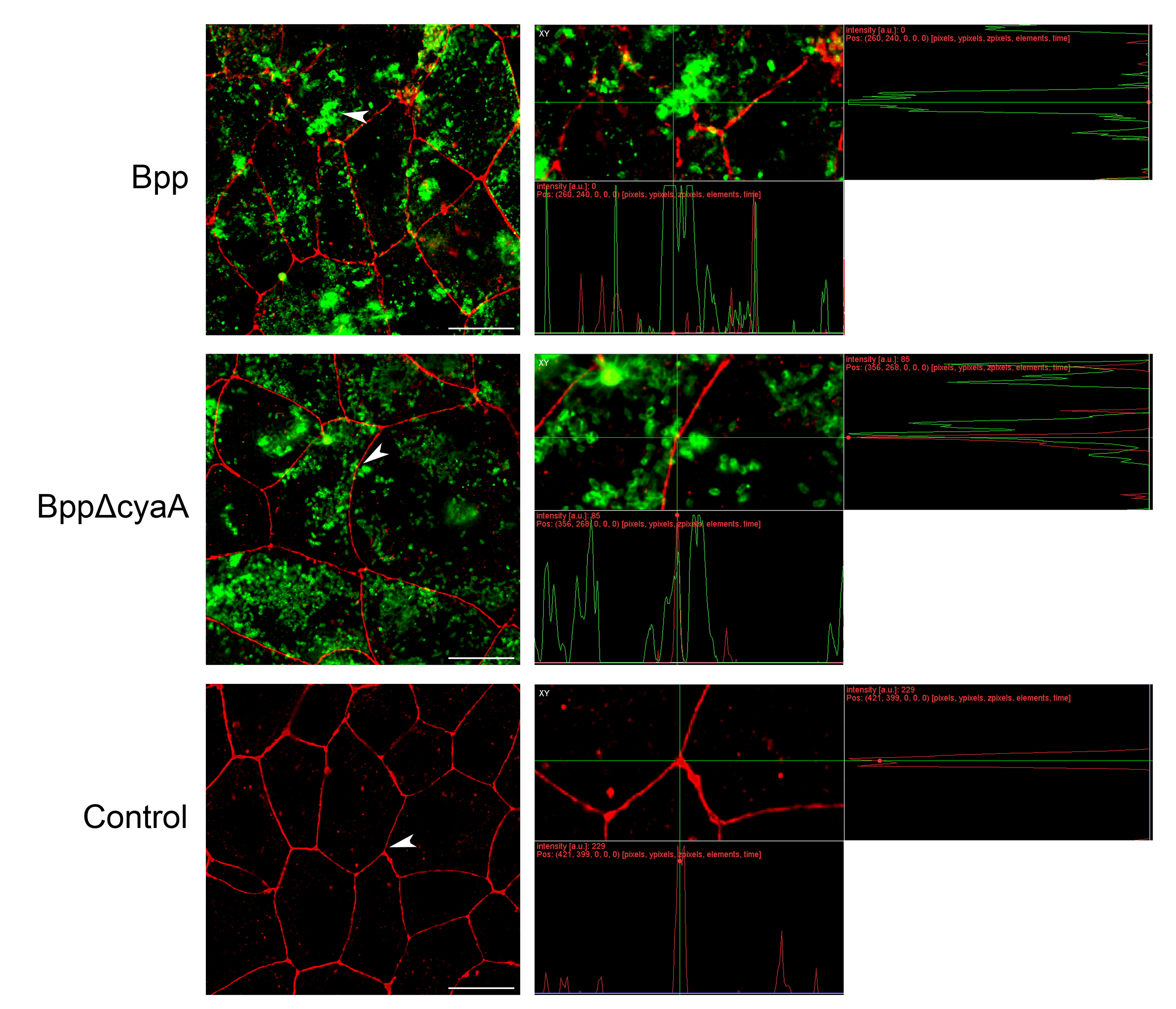

Supplement: S1 Fig — Seven-day-old polarized monolayers incubated with wild type B. parapertussis (Bpp) (MOI 1), a CyaA deficient mutant (BppΔCyaA) (MOI 1), or medium alone (control) for 24 h at 37°C, were washed, fixed and permeabilized prior to labeling tight junctions protein occludin (red) and cell-associated bacteria (green). Fluorescence microscopy images were taked at 24 hours post-infection and analyzed using the ImageJ plugin "view 5D" to generate overlay charts from the sum intensity x- and y-projection, combining histogram information from the red (occludin) and green (cell-associated bacteria) channels at specific xy points. Scale bar: 10 μm. (TIF) [file pone.0291331.s001.tif]
